# The Brain–Atrial Fibrillation–Recent Rehabilitation Axis: A Modern Approach

**DOI:** 10.3390/healthcare14060765

**Published:** 2026-03-18

**Authors:** Aleksandra Maria Piotrowska, Kamil Salwa, Karol Kazirod-Wolski, Janusz Sielski

**Affiliations:** Collegium Medicum, Jan Kochanowski University, 25-369 Kielce, Poland; alemarpiotrowska@gmail.com (A.M.P.); kamil.salwa@gmail.com (K.S.); kk-wolski@wp.eu (K.K.-W.)

**Keywords:** atrial fibrillation, ischemic stroke, anticoagulation, neurorehabilitation, hemodynamic instability, Heart–Brain Team

## Abstract

Atrial fibrillation (AF) is the most common sustained cardiac arrhythmia and a major contributor to ischemic stroke, heart failure, hospitalization, and mortality. AF-related strokes account for approximately 20–30% of all ischemic strokes and are typically associated with more severe neurological deficits and poorer long-term outcomes. As the prevalence of AF continues to rise with population aging, optimizing both stroke prevention and post-stroke management has become increasingly important. This narrative review summarizes current evidence on AF in the context of ischemic stroke, with particular emphasis on anticoagulation, acute stroke management, and early neurorehabilitation. Special attention is given to the unique challenges of AF-associated stroke, including hemodynamic instability, recurrent embolic risk, bleeding risk during anticoagulation, and the need for individualized rehabilitation strategies. We also discuss interdisciplinary care models, including the Hemodynamic Gating Matrix and the Heart–Brain Team approach, as potential frameworks for integrating cardiovascular and neurological management during recovery. AF-related stroke requires coordinated care across cardiology, neurology, and rehabilitation medicine. A physiology-guided and interdisciplinary approach may improve functional recovery while maintaining cardiovascular safety in this high-risk population.

## 1. Introduction and Clinical Relevance

Atrial fibrillation (AF) is the most common sustained supraventricular arrhythmia and one of the major challenges in contemporary cardiology [1,2,3]. Over recent decades, AF has been referred to as an “epidemic of the 21st century,” reflecting both the rapid increase in the number of affected individuals and the growing clinical burden driven by severe thromboembolic complications, particularly ischemic stroke [2,3,4]. AF is also associated with an increased risk of heart failure, more frequent hospitalizations, impaired quality of life, and higher all-cause mortality [1,3].

Advances in cardiovascular care have prolonged life expectancy; however, they have also highlighted new challenges related to chronic diseases of older age, among which AF occupies a prominent position [2,3]. AF rarely occurs as an isolated condition; it most often coexists with other cardiovascular disorders, creating a complex clinical phenotype that requires a multifaceted diagnostic and therapeutic approach [1].

The clinical relevance of AF largely stems from its complications. It is estimated that AF accounts for approximately 20–30% of all ischemic strokes, and AF-related strokes are characterized by a more severe clinical course, greater neurological deficit, and poorer long-term outcomes [4]. In addition, the presence of AF is associated with an increased risk of all-cause death, independent of other cardiovascular risk factors [1,3].

## 2. Methodology

This article was designed as a comprehensive narrative review synthesizing current evidence at the intersection of cardiology, neurology, and rehabilitation medicine. To ensure methodological rigor and reproducibility, a structured literature search was conducted adhering to the fundamental principles of the Preferred Reporting Items for Systematic Reviews and Meta-Analyses (PRISMA) statement, adapted for a narrative synthesis. The primary objective was to integrate data concerning atrial fibrillation pathophysiology with stroke mechanisms and subsequent neurorehabilitation strategies.

A systematic search was performed across PubMed/MEDLINE, Scopus, and the Cochrane Library databases, covering the period from January 2020 to January 2026. The search strategy employed a combination of Medical Subject Headings (MeSH) and free-text terms relevant to three core domains: atrial fibrillation management, cardioembolic stroke pathophysiology, and early post-stroke mobilization. Boolean operators were utilized to refine the results, specifically targeting the intersection of hemodynamic stability and rehabilitation outcomes as detailed in the full search string provided in Appendix A.

**Figure 1:**
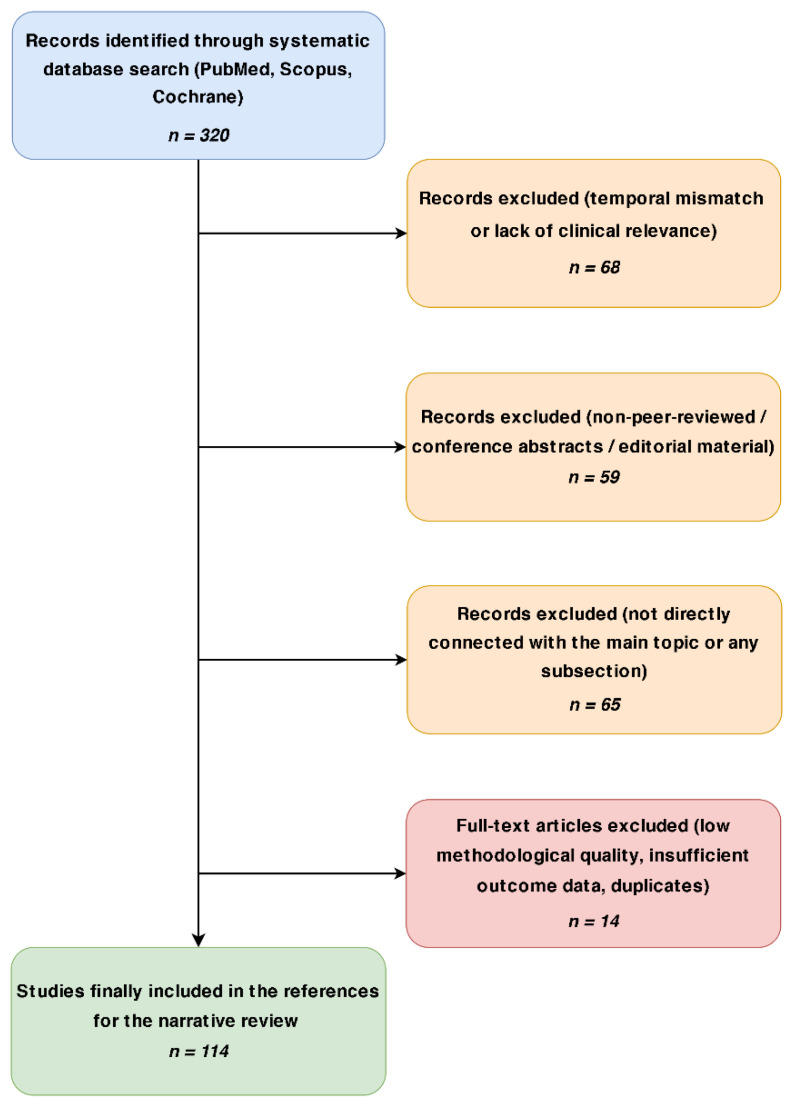
Algorithm of study selection process for the narrative review. Arrows indicate the flow of study selection. Blue box represent identified records, orange boxes indicate excluded records during screening, red boxes represent excluded full-text articles, and the green box indicates the final number of included studies.

The initial search yielded a total of 320 records. In the first phase of selection, 45 duplicates were identified and removed. The remaining 275 records underwent screening based on titles and abstracts. At this stage, 125 articles were excluded due to lack of direct relevance to the interdisciplinary scope of the review, specifically those focusing solely on isolated surgical techniques or general cardiology without neurological or rehabilitative implications. The remaining 150 full-text articles were assessed for eligibility. During this final appraisal, 36 records were excluded based on the following criteria: study design limitations (e.g., case reports, small case series, or editorials lacking original data), insufficient methodological rigor, publication in languages other than English, or lack of access to the full text. Ultimately, 114 references were selected for inclusion in this review to support the conceptual framework of the Hemodynamic Gating Matrix and the Heart–Brain Team model. The detailed inclusion and exclusion criteria applied during the literature screening are presented in Table 1, and the overall study selection process is illustrated in the PRISMA flow diagram in Figure 1.

## 3. Epidemiology of Atrial Fibrillation and the Impact of Population Aging

The prevalence of AF has been steadily increasing worldwide [2,3], with a lifetime risk of approximately 20–25% after the age of 40 years [5]. Population aging is the principal determinant of this trend [2,3]. Older individuals accumulate comorbidities such as hypertension, heart failure, type 2 diabetes, and chronic kidney disease, which increase both AF incidence and the proportion of persistent and long-standing persistent forms [1,3].

## 4. In-Hospital Management of Atrial Fibrillation: Acute-Phase Treatment and Anticoagulant Prophylaxis

Hospitalization of patients with atrial fibrillation is most often related to acute arrhythmia onset, severe symptoms, hemodynamic instability, or the need for diagnostic evaluation and treatment modification. Acute management focuses on stabilization of the patient’s clinical condition, rhythm or ventricular rate control, and prevention of thromboembolic complications.

Anticoagulation therefore represents a cornerstone of stroke prevention in AF. The decision to initiate therapy is guided by thromboembolic risk assessment using the CHA_2_DS_2_-VA score, while bleeding risk should be evaluated using tools such as HAS-BLED to identify and modify reversible risk factors rather than to withhold treatment [6,7,8,9,10,11].

## 5. Outpatient Treatment and Long-Term Care

Long-term management of atrial fibrillation is based on an integrated care model focusing on stroke prevention, symptom control, and modification of cardiovascular risk factors, as recommended by current European Society of Cardiology guidelines [1,12]. Older patients frequently present with frailty and multimorbidity, which complicate therapeutic decision making and require individualized assessment when considering anticoagulation or rhythm-control strategies [13,14]. Continuity of care, including collaboration between primary care physicians and cardiologists, is essential for ensuring treatment safety and improving long-term clinical outcomes [15,16].

## 6. Atrial Fibrillation and Ischemic Stroke: Identifying and Addressing Critical Gaps in the Management Pathway

Atrial fibrillation (AF) remains the strongest independent risk factor for thromboembolic events, primarily due to thrombus formation within the left atrial appendage [17,18]. In the absence of anticoagulation, AF increases the risk of ischemic stroke approximately fivefold [4], with risk modulated by age, sex, and comorbidities. Thromboembolic risk assessment was initially standardized using the CHA_2_DS_2_-VASc score [6], while the simplified CHA_2_DS_2_-VA score has recently gained acceptance without loss of predictive performance [19]. Stroke prevention relies on VKAs and NOACs, which demonstrate comparable efficacy; however, NOACs have a more favorable safety profile, with lower rates of intracranial and gastrointestinal bleeding [20]. VKAs remain mandatory in selected populations, including patients with mechanical heart valves, moderate-to-severe mitral stenosis, and those on dialysis [21].

In acute ischemic stroke presenting within 4.5 h, eligibility for reperfusion therapy depends largely on prior anticoagulation. Intravenous thrombolysis is generally not recommended in patients who received NOACs within 48 h unless low anticoagulant activity is documented (anti–factor Xa < 0.5 U/mL or thrombin time < 60 s). In dabigatran-treated patients, idarucizumab followed by thrombolysis is preferred, whereas andexanet alfa is not recommended prior to thrombolysis in factor Xa inhibitor users. In patients receiving VKAs, thrombolysis is permitted if INR ≤ 1.7 and contraindicated above this threshold [22].

Mechanical thrombectomy (MT) is a cornerstone therapy for large vessel occlusion in the anterior circulation. Within 6 h of symptom onset, MT combined with best medical management (including thrombolysis when indicated) significantly improves functional outcomes compared with medical therapy alone. In the 6–24 h window, MT is recommended in selected patients meeting DEFUSE-3 or DAWN criteria based on advanced imaging demonstrating salvageable tissue. When both thrombolysis and MT are indicated, a bridging strategy should be used without delaying either treatment [23].

Patients undergoing successful pulmonary vein isolation (PVI) represent a distinct group. Anticoagulation is recommended for at least two months post ablation, with long-term continuation guided by individual thromboembolic risk (CHA_2_DS_2_-VA) rather than rhythm outcome alone [21]. The ALONE-AF trial demonstrated that discontinuation of NOAC therapy after 12 months without arrhythmia recurrence reduced net clinical events (0.3% vs. 2.2%) [24]. Similarly, the OCEAN study found no difference in thromboembolic outcomes between rivaroxaban and aspirin after successful ablation performed at least one year earlier, suggesting that effective PVI may sufficiently reduce stroke risk in selected low-to-moderate-risk patients (CHA_2_DS_2_-VASc 1–3) [25].

Stroke risk is transiently elevated after elective cardioversion, with a sevenfold increase during the first two weeks irrespective of anticoagulant type or baseline risk factors [26]. In contrast, emergency department cardioversion for recent-onset AF (<48 h, or <7 days with adequate anticoagulation) has been associated with a very low 30-day rate of stroke or death (approximately 0.37%) [27]. Current guidelines limit cardioversion without effective anticoagulation to within 24 h of AF onset [21].

Silent atrial fibrillation, or atrial high-rate episodes (AHREs), detected by implantable or wearable devices, remains clinically challenging. Despite the absence of symptoms, AHRE is associated with an approximately 2.5-fold increase in stroke risk [28]. The ARTESiA trial demonstrated a 37% reduction in stroke or systemic embolism with apixaban compared with aspirin in subclinical AF, at the cost of increased major bleeding, predominantly gastrointestinal [29]. Conversely, NOAH-AFNET 6 suggested that routine anticoagulation in very short episodes (<24 h) may not provide net benefit, except possibly in secondary prevention populations [30].

Given bleeding concerns with long-term anticoagulation, novel agents targeting factor XIa aim to reduce thrombosis while minimizing interference with physiological hemostasis [31,32]. In AZALEA-TIMI 71, abelacimab reduced major bleeding by over 60% compared with rivaroxaban [33] and is under investigation in high bleeding-risk and oncology populations, including settings of combined antithrombotic therapy [34]. Asundexian failed to demonstrate sufficient efficacy in OCEANIC-AF despite favorable safety [35], although further evaluation in secondary prevention is ongoing following PACIFIC-STROKE [36]. Further studies are underway to improve efficacy in secondary stroke prevention in the OCEANIC STROKE trial [37]. Milvexian may offer improved efficacy, but final results from LIBREXIA-AF are pending [38].

## 7. Early Neurorehabilitation Programs and Protocols in Brain Stroke Connected to Atrial Fibrillation

A quantitative synthesis of the key clinical studies supporting these conceptual frameworks—including sample sizes and intervention protocols—is presented in Table 2.

### 7.1. Pathophysiological Basis: The Hemodynamic–Metabolic Mismatch

The clinical and rehabilitative management of acute ischemic stroke secondary to AF represents one of the most demanding domains in contemporary vascular neurology and rehabilitation medicine. It is important to emphasize that the protocols presented herein, including the ‘Hemodynamic Gating Matrix’ and ‘Rehab-Sync’, represent a theoretical translational synthesis. They are derived from the extrapolation of established cardiovascular guidelines [21] and general stroke rehabilitation evidence (AVERT trial), rather than direct Randomized Controlled Trials specifically designed for the AF-stroke population.

Unlike atherothrombotic or small-vessel strokes, cardioembolic events related to atrial fibrillation emerge from a systemic cardiovascular disorder that directly shapes cerebral perfusion dynamics before, during, and after the ischemic insult. As a result, the neurological deficit observed at presentation reflects not only focal tissue loss but also a broader disruption of cerebrovascular autoregulation and metabolic homeostasis, which together define the trajectory of recovery [49].

Patients with AF-associated stroke consistently demonstrate more severe initial neurological deficits and worse early outcomes. This observation is not solely attributable to infarct size, although large-vessel occlusions within the anterior circulation are common. It also reflects the pre-existing vulnerability of the atrial fibrillation brain, which is frequently characterized by chronic hypoperfusion, diffuse white matter injury, and impaired vasoreactivity. These background alterations constrain adaptive neuroplastic mechanisms at the very moment when they are most critically needed. Consequently, early neurorehabilitation in this population cannot be conceptualized as a standardized extension of acute stroke care but must instead be viewed as a precision intervention tightly coupled to cardiovascular physiology [50].

The pathophysiological foundation of atrial fibrillation-related stroke rehabilitation rests on the concept of the hemodynamic–metabolic mismatch. Chronic irregular ventricular rhythm produces beat-to-beat variability in cardiac output, resulting in unstable cerebral blood flow even in the absence of overt hypotension. Over time, this instability contributes to microvascular rarefaction and leukoaraiosis, particularly within watershed territories. When an acute embolic occlusion occurs, the ischemic penumbra that surrounds the infarct core is therefore larger, more fragile, and less tolerant of systemic perturbations. Early rehabilitation strategies must acknowledge that even modest fluctuations in blood pressure, heart rate, or autonomic tone may translate into clinically meaningful changes in tissue perfusion [42].

### 7.2. Mobilization Readiness and the Hemodynamic Gating Matrix

In the hyperacute phase following cardioembolic stroke, typically encompassing the first forty-eight hours, the primary objective of rehabilitation is preservation rather than restoration. During this interval, the penumbral tissue remains metabolically active but functionally suppressed, relying on tenuous collateral flow. Excessive physical stimulation or premature verticalization may provoke abrupt changes in cerebral perfusion pressure, leading to irreversible infarct expansion. At the same time, prolonged immobilization is associated with its own spectrum of complications, including atelectasis, aspiration pneumonia, venous thromboembolism, and rapid musculoskeletal deconditioning. The challenge for the rehabilitation team lies in navigating between these competing risks without compromising neurological stability [51].

The evolution from time-based to physiology-based mobilization represents a critical paradigm shift in this context. Traditional stroke protocols frequently recommended mobilization within a fixed temporal window, often twenty-four hours after symptom onset, based on population-level benefits observed in heterogeneous stroke cohorts. However, such an approach fails to account for the unique cardiovascular instability inherent to atrial fibrillation. Contemporary models instead emphasize readiness for mobilization as a dynamic state determined by measurable physiological parameters rather than elapsed time alone [52].

The Hemodynamic Gating Matrix has emerged as a practical framework for operationalizing this philosophy. This model integrates continuous cardiac monitoring, blood pressure assessment, and autonomic testing to determine whether a patient is physiologically prepared for progressive activity. Cardiac reserve is evaluated through sustained ventricular rate control without episodes of Rapid Ventricular Response during rest or minimal exertion. Autonomic integrity is assessed via orthostatic challenges, ensuring that head-of-bed elevation does not precipitate clinically significant hypotension or tachycardia. Rhythm stability is confirmed using real-time telemetry to exclude arrhythmic deterioration during therapeutic maneuvers. Only when these criteria are met is advancement toward sitting, standing, or ambulation considered appropriate [53].

The relevance of this approach is underscored by the phenomenon commonly referred to as stroke–heart interaction. Acute cerebral injury, particularly when involving the insular cortex, can itself provoke autonomic imbalance and exacerbate pre-existing atrial fibrillation. This bidirectional relationship creates a feedback loop in which neurological injury destabilizes cardiac rhythm, which in turn further compromises cerebral perfusion. Early rehabilitation conducted without awareness of this interaction risks amplifying rather than mitigating systemic instability. Continuous monitoring and close interdisciplinary communication are therefore indispensable components of safe mobilization strategies [54].

While conventional stroke rehabilitation predominantly relies on time-dependent protocols, such generalized approaches fail to accommodate the beat-to-beat cerebral perfusion instability inherent to atrial fibrillation. Given the profound lack of targeted clinical guidelines for this cohort, we specifically focused on the Hemodynamic Gating Matrix and the Brain–Heart Team model as synthesized conceptual frameworks driving a paradigm shift toward physiology-based gating. These specific theoretical models were selected because they uniquely mandate continuous autonomic validation prior to verticalization, although they remain proposed paradigms necessitating formal validation in future dedicated prospective trials.

### 7.3. Anticoagulation Considerations and Pharmacological Synchronization

Anticoagulation represents another axis along which early rehabilitation must be carefully calibrated. The introduction of direct oral anticoagulants has transformed secondary prevention in atrial fibrillation-related stroke, allowing earlier initiation of therapy with an acceptable safety profile. Large contemporary trials have demonstrated that, when appropriately selected, patients may begin anticoagulation within days of the index event without a prohibitive increase in hemorrhagic complications [39]. Nevertheless, from a rehabilitative standpoint, anticoagulation introduces additional considerations that extend beyond the binary decision of when to start treatment.

The pharmacokinetics of direct oral anticoagulants are characterized by predictable absorption and defined peak plasma concentrations. Periods of maximal drug activity may coincide with heightened vulnerability to bleeding, particularly in the setting of exercise-induced hypertension or falls during therapy. The Rehab-Sync concept addresses this issue by aligning the intensity of rehabilitation sessions with the pharmacological profile of the anticoagulant. High-demand activities such as gait training or robotic-assisted movements are preferentially scheduled during trough or mid-range drug levels, reducing theoretical bleeding risk while preserving opportunities for intensive practice [40].

This synchronization requires meticulous coordination among physicians, nurses, and therapists. Medication administration times must be clearly communicated, and daily therapy schedules adjusted accordingly. Such coordination exemplifies the broader need for an integrated Brain–Heart Team Model in the management of atrial fibrillation-related stroke. Rehabilitation is no longer an isolated downstream process but a core component of secondary prevention and systemic stabilization [55,56].

Despite these precautions, the early post-stroke period remains one of heightened risk for both hemorrhagic transformation and recurrent embolization. This dual threat necessitates a low threshold for interrupting therapy in response to neurological change. Even subtle alterations in mental status, motor strength, or autonomic parameters may herald intracranial bleeding or new ischemia. Protocols emphasizing rapid recognition and immediate neuroimaging are essential to ensure that rehabilitation enhances rather than jeopardizes patient outcomes [57].

### 7.4. Cognitive–Motor Interference and Dual-Task Strategies

Beyond hemodynamic and pharmacological considerations, atrial fibrillation-related stroke imposes a distinctive cognitive burden that profoundly influences rehabilitation trajectories. Atrial fibrillation is independently associated with accelerated cognitive decline, even in the absence of overt stroke. Mechanisms proposed to underlie this association include chronic cerebral hypoperfusion, silent micro-emboli, and systemic inflammation. Following an acute stroke, these pre-existing vulnerabilities manifest as disproportionate impairments in executive function, attention, and processing speed relative to the severity of focal motor deficits.

During early rehabilitation, such cognitive impairments give rise to cognitive–motor interference, a phenomenon in which the neural resources required for movement compete with those necessary for cognitive control. Clinically, this may present as instability during ambulation when the patient is asked to perform a simple mental task or engage in conversation. The risk of falls and secondary injury is particularly concerning in anticoagulated individuals, amplifying the consequences of this interference [46].

To address this challenge, modern rehabilitation paradigms increasingly emphasize simultaneous dual-task training. Rather than postponing cognitive engagement until later stages of recovery, patients are exposed to controlled combinations of motor and cognitive demands once physiological stability is established. Examples include performing working memory tasks during treadmill walking or verbal fluency exercises during balance training. This approach is designed to promote more efficient integration of cognitive and motor networks, reflecting the demands of real-world function rather than the artificial separation imposed by traditional therapy models [48].

### 7.5. Cognitive Preservation, Screening, and Tailored Rehabilitation Strategies

While the association between atrial fibrillation and accelerated cognitive decline is well-documented, recent systematic reviews emphasize that the trajectory of neurodegeneration is modifiable through aggressive upstream management. Meta-analyses by Lee et al. and Agarwal et al. provide compelling evidence that adequate oral anticoagulation significantly mitigates the risk of cognitive impairment (by approximately 29%) compared to placebo or antiplatelet therapy, likely by preventing silent cerebral micro-infarctions [58,59]. Consequently, the Brain–Heart Team Model prioritizes the optimization of the ABC pathway (Atrial fibrillation Better Care)—specifically strict anticoagulation adherence and rigorous control of cardiovascular comorbidities (hypertension, diabetes)—as the foundational strategy for cognitive preservation [60]. Furthermore, consistent with Canadian Stroke Best Practice Recommendations, our framework mandates routine cognitive screening (e.g., using the Montreal Cognitive Assessment) for all AF-stroke survivors to identify domain-specific deficits early [61]. This diagnostic step enables the transition from generic interventions to “tailored” cognitive rehabilitation, targeting executive dysfunction and attention deficits specific to the patient’s profile. Only upon this foundation should advanced paradigms such as dual-task training be introduced; while evidence supports its efficacy in reducing cognitive–motor interference, recent randomized trials suggest its benefits are maximized when implemented as a graded protocol following the stabilization of basic cognitive and motor reserves [62].

### 7.6. Neuromodulation and Safety in Patients with Cardiac Devices

Neuromodulation has gained prominence as an adjunctive strategy for enhancing neuroplasticity in this setting. Vagus nerve stimulation, when paired with task-specific motor practice, has demonstrated efficacy in improving upper-limb function after ischemic stroke. Its relevance to atrial fibrillation-related stroke extends beyond motor outcomes. By activating the cholinergic anti-inflammatory pathway, vagus nerve stimulation may attenuate the systemic and central inflammatory responses that are particularly pronounced in atrial fibrillation, thereby fostering a more favorable environment for neural remodeling [47].

Similarly, transcranial direct current stimulation has been employed to modulate cortical excitability and interhemispheric balance following large cortical infarcts. By priming the ipsilesional motor cortex before therapy sessions, this technique aims to enhance the efficiency of subsequent practice. In patients with atrial fibrillation, however, the widespread presence of implanted cardiac devices necessitates rigorous safety protocols. Continuous monitoring and adherence to updated guidelines are essential to prevent adverse interactions between neuromodulatory currents and cardiac hardware [63].

While the efficacy of non-invasive brain stimulation (NIBS) strategies, including transcranial direct current stimulation (tDCS) and vagus nerve stimulation (VNS), is increasingly established in general ischemic stroke cohorts—demonstrating tangible improvements in motor learning and upper-limb functional recovery [47,55]—it is imperative to distinguish these generalized findings from the specific pathophysiological context of atrial fibrillation-associated stroke. To date, no large-scale Randomized Controlled Trials have exclusively isolated the AF-stroke subpopulation to validate these modalities; thus, current applications in this group rely largely on pathophysiological extrapolation rather than direct evidence.

This extrapolation warrants extreme caution given the high prevalence of cardiac implantable electronic devices (CIEDs), such as pacemakers and implantable cardioverter-defibrillators, among patients with AF. Although recent safety guidelines suggest that tDCS can be theoretically administered in patients with bipolar sensing pacemakers under strict monitoring [63], the electromagnetic field generated by tDCS or VNS carries a theoretical risk of interference with CIED sensing algorithms or pacing thresholds, potentially precipitating inappropriate shocks or inhibition of pacing. Furthermore, the autonomic modulation inherent to VNS—specifically its parasympathetic activation via the cholinergic anti-inflammatory pathway—could unpredictably alter atrial refractoriness in patients with paroxysmal AF, theoretically influencing arrhythmogenesis [54]. Consequently, until dedicated safety data for the AF-CIED population becomes available, neuromodulation should be restricted to patients without contraindications or conducted within strictly controlled clinical trial settings involving continuous telemetric surveillance.

### 7.7. Technological Support: Wearables and AI Analytics

Technological advances have further expanded the scope of early rehabilitation in atrial fibrillation-related stroke. Wearable sensors capable of monitoring movement, heart rate, and autonomic responses in real time provide unprecedented insight into the physiological cost of activity. These data allow therapists to titrate exercise intensity dynamically, preventing overexertion and identifying early signs of cardiovascular or neurological stress. Such systems transform rehabilitation from a static prescription into a responsive process guided by continuous feedback [64].

AI-based analytics build upon this foundation by integrating multimodal data into predictive models of recovery. Early patterns of movement efficiency, cardiovascular response, and cognitive engagement can be analyzed to forecast functional trajectories and identify patients at risk for stagnation or decline. In atrial fibrillation-stroke survivors, where recovery is often nonlinear and punctuated by medical complications, such predictive capacity holds particular promise for personalizing therapy intensity and timing [65].

### 7.8. Systemic Integration and the Heart–Brain Team Model

At the systemic level, nutritional and metabolic considerations further influence rehabilitation outcomes. Older patients with atrial fibrillation frequently exhibit sarcopenia and frailty, conditions exacerbated by acute illness and immobility. Early nutritional optimization, including adequate protein intake and attention to inflammatory status, supports muscle preservation and enhances responsiveness to therapy. While not a primary focus of neurorehabilitation protocols, these measures contribute meaningfully to overall recovery capacity [66].

The complexity of early rehabilitation in atrial fibrillation-related stroke underscores the necessity of interdisciplinary integration. Neurological recovery, cardiovascular stability, cognitive function, and systemic health are inseparable components of a single clinical trajectory. Effective rehabilitation therefore demands a coordinated strategy in which each therapeutic decision is informed by its potential impact across these domains. The Brain–Heart Team Model embodies this integration, aligning expertise from neurology, cardiology, rehabilitation medicine, and allied health disciplines toward a unified goal of functional restoration [56].

As evidence continues to accumulate, it is increasingly clear that early neurorehabilitation in atrial fibrillation-associated stroke cannot rely on generic principles derived from mixed stroke populations. Instead, it requires a tailored approach grounded in physiological monitoring, pharmacological awareness, cognitive integration, and technological support. Such an approach recognizes that recovery of function is inseparable from maintenance of systemic stability and that meaningful rehabilitation begins not after medical management has concluded but alongside it, from the earliest stages of care [67].

### 7.9. Monitoring Physiological Fatigue and Autonomic Balance

This reconceptualization of early rehabilitation as an integrated, physiology-sensitive process has significant implications for outcome measurement and clinical decision making. Traditional rehabilitation metrics have focused predominantly on motor scales and activities of daily living assessed at discrete time points. In atrial fibrillation-related stroke, however, recovery unfolds along a multidimensional axis in which cardiovascular stability, cognitive resilience, and autonomic adaptability exert continuous influence on functional gains. Consequently, early progress may be nonlinear, with transient plateaus or regressions reflecting systemic perturbations rather than failure of neuroplastic mechanisms. Recognizing this pattern is essential to avoid premature escalation or inappropriate withdrawal of rehabilitative interventions [68].

Within this framework, careful attention must be paid to the concept of physiological fatigue. In patients with atrial fibrillation, the metabolic cost of movement is often disproportionately high due to inefficient cardiac output and impaired peripheral oxygen delivery. Activities that appear modest from a biomechanical perspective may therefore impose substantial cardiovascular strain. Early rehabilitation protocols must incorporate mechanisms to detect and respond to such fatigue before it manifests as overt decompensation. Continuous monitoring of heart rate variability, perceived exertion, and recovery kinetics after activity provides valuable insight into an individual patient’s tolerance and adaptive capacity [43].

The role of heart rate variability as a surrogate marker of autonomic balance has gained increasing recognition in this context. Reduced variability reflects diminished parasympathetic tone and heightened sympathetic dominance, both of which are common in atrial fibrillation and further accentuated after stroke. Persistently low variability during therapy sessions may signal inadequate recovery or excessive stress, prompting adjustment of intensity or timing. Conversely, gradual normalization of variability over successive sessions may indicate improving autonomic integration and readiness for more demanding tasks [44].

### 7.10. Robotic Rehabilitation and Prevention of Learned Nonuse

Another critical dimension of early rehabilitation in this population is the prevention of learned nonuse. Large cortical infarcts frequently result in profound initial weakness, particularly of the upper limb. When combined with cognitive impairment and fatigue, this weakness predisposes patients to compensatory strategies that limit engagement of the affected limb. Early introduction of task-specific training, supported by neuromodulation or robotic assistance when appropriate, is therefore essential to counteract maladaptive plasticity. In atrial fibrillation-related stroke, such interventions must be delivered within the constraints imposed by cardiovascular safety, reinforcing the importance of individualized dosing rather than rigid schedules.

The integration of robotics into early rehabilitation offers particular advantages in this regard. Robotic devices enable high-repetition, precisely controlled movements with adjustable assistance, allowing patients to engage in intensive practice without excessive cardiovascular load. When coupled with real-time monitoring, these systems can automatically modulate resistance or support in response to physiological signals, maintaining activity within a defined safety envelope. This capability is especially valuable in patients with fluctuating tolerance due to arrhythmia or autonomic instability [69].

### 7.11. Implementation Challenges and Continuum of Care

Cognitive engagement during early rehabilitation also warrants nuanced consideration. While dual-task training has demonstrated benefits in mitigating cognitive–motor interference, its implementation must be carefully staged. Excessive cognitive demand imposed too early may exacerbate fatigue or precipitate disengagement, particularly in patients with pre-existing executive dysfunction. A graded approach, beginning with low-complexity cognitive tasks integrated into simple motor activities and progressing as stability improves, aligns with principles of motor learning and respects the limited processing capacity characteristic of the early post-stroke period [70].

Social and environmental factors further shape the effectiveness of early rehabilitation. Hospital settings often impose sensory deprivation or overload, both of which can negatively influence cognitive recovery. Structuring therapy sessions to approximate real-world contexts, while maintaining safety, may enhance transfer of skills and reinforce adaptive neural networks. For atrial fibrillation–stroke survivors, whose return to community living is often complicated by fear of recurrence or bleeding, early exposure to controlled but meaningful activities may also confer psychological benefits that support long-term adherence to rehabilitation.

As patients transition from the acute care environment to inpatient or home-based rehabilitation, continuity of the Brain–Heart Team Model becomes increasingly important. Disruptions in monitoring, medication timing, or therapy coordination during this transition may negate gains achieved in the hospital. Tele-rehabilitation platforms and wearable monitoring devices offer a means of extending physiological oversight beyond institutional boundaries, ensuring that exercise intensity remains aligned with cardiovascular status and anticoagulation schedules. Such continuity is particularly relevant given the paroxysmal nature of atrial fibrillation, which may recur unpredictably during the subacute phase [71].

From a systems perspective, implementation of these advanced rehabilitation paradigms requires institutional commitment and interdisciplinary training. Therapists must be proficient not only in motor rehabilitation techniques but also in interpretation of cardiovascular data and recognition of arrhythmic or autonomic warning signs. Similarly, physicians must appreciate the rehabilitative implications of pharmacological decisions, including rate control strategies and anticoagulant selection. Shared protocols and regular communication are essential to harmonize these perspectives and translate evidence into consistent practice.

**Figure 2:**
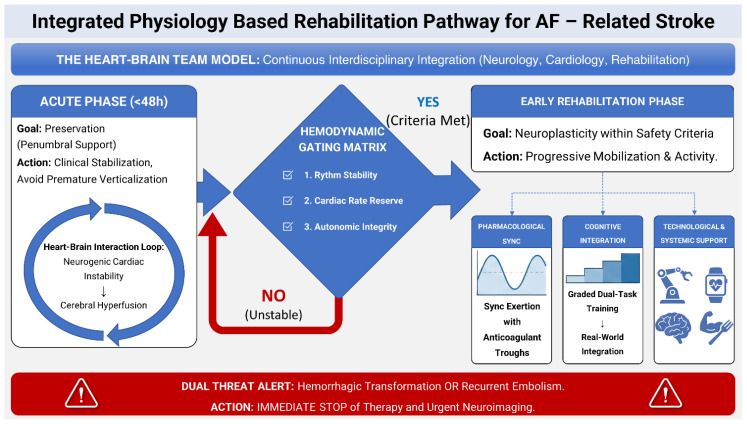
Operational schematic of the Hemodynamic Gating Matrix. Note: This flow-chart illustrates the theoretical decision-making pathway. The central diamond blocks represent the sequential physiological safety checks (rhythm stability → rate reserve → autonomic integrity) required before patient mobilization [21,39,40,43,44,54,72]. Arrows indicate the direction of clinical decision flow; the red pathway represents failure to meet stability criteria and the need to return to the acute stabilization phase.

Future directions in the field are likely to focus on further personalization of early rehabilitation through biomarker integration. Genetic variability influencing anticoagulant metabolism, inflammatory response, or neurotrophic factor expression may eventually inform individualized timing and intensity of therapy. While such approaches remain investigational, they underscore the trajectory toward increasingly precise alignment of rehabilitation with biological context. In atrial fibrillation-related stroke, where heterogeneity of presentation and response is the rule rather than the exception, this precision holds particular promise [56]. The operational architecture of this precision-medicine approach, delineating the bidirectional interplay between the Hemodynamic Gating Matrix, pharmacological synchronization, and the interdisciplinary Brain–Heart Team Model, is illustrated in Figure 2.

### 7.12. The Physiological Safety Limits: Reconciling Early Mobilization with AVERT Trial Data

The implementation of early rehabilitation in this high-risk cohort must be critically contextualized against the findings of the Phase III AVERT trial, which suggested that high-dose mobilization within 24 h could reduce the odds of a favorable outcome [73]. However, a nuanced interpretation of these results is essential. As rigorously argued by Luft and Kesselring, the adverse outcomes observed in AVERT were likely not attributable to mobilization per se, but rather to the trial’s pragmatic design, which prioritized a rigid chronological target over physiological stability, thereby failing to prevent episodes of significant hypotension during verticalization [74]. Our framework directly addresses this methodological gap by shifting the paradigm from “time-dependent” to “physiology-dependent” mobilization. The Hemodynamic Gating Matrix operationalizes the safety concerns raised by Luft and Kesselring, prohibiting mobilization in patients exhibiting hemodynamic intolerance (e.g., Orthostatic Hypotension or Rapid Ventricular Response). By enforcing strict “Stop-Signal” criteria, our model ensures that the penumbra is protected from hypoperfusion, effectively decoupling the benefits of early activity from the risks of hemodynamic instability identified in the AVERT cohort [41,75].

### 7.13. Operationalization of the Hemodynamic Gating Matrix: Thresholds and Safety Criteria

To mitigate the risk of hypoperfusion in the penumbral tissue, the Hemodynamic Gating Matrix enforces stricter physiological boundaries than standard intensive care mobilization protocols. While conventional stroke algorithms, such as those utilized in the AVERT trial, permit mobilization within broad hemodynamic ranges (systolic blood pressure: 110–220 mmHg; heart rate: 40–110 bpm) [76], this framework defines readiness through a tighter, physiology-based “safety envelope” tailored to the autonomic instability inherent to AF [21,77]. Specifically, mobilization is contraindicated if any of the following Stop-Signal Criteria are met:

- **Chronotropic Incompetence or Instability:** Resting ventricular rate >100 bpm or an exertional increase to >110 bpm (Rapid Ventricular Response), reflecting the upper limit of lenient rate control recommended by the 2024 ESC Guidelines [21] to preserve diastolic filling time.
- **Orthostatic Intolerance:** A sustained reduction in SBP ≥ 20 mmHg or diastolic BP ≥ 10 mmHg within 3 min of verticalization (classic Orthostatic Hypotension), or the emergence of Postural Orthostatic Tachycardia (increment >30 bpm), both of which are independent predictors of poor functional outcomes in the subacute stroke phase.
- **Blood Pressure Variability (BPV):** Exceeding a functional limit of >15% coefficient of variation in SBP during session monitoring, as recent evidence (2025) correlates high BPV with infarct expansion and impaired cerebral autoregulation [78,79].

Unlike standard ICU criteria, which prioritize Mean Arterial Pressure (MAP) > 65 mmHg largely for systemic organ perfusion, the Gating Matrix treats asymptomatic arrhythmia recurrence and occult orthostasis as absolute barriers to verticalization, prioritizing cerebral autoregulatory reserve over functional activation [41]. The precise physiological readiness thresholds, specific interruption signals, and the progressive mobilization escalation pathways constituting the Hemodynamic Gating Matrix are comprehensively detailed in Table 3.

In summary, early neurorehabilitation after AF-associated stroke represents a complex, high-stakes endeavor that extends well beyond conventional motor training. The distinctive pathophysiological substrate of the atrial fibrillation brain, characterized by hemodynamic instability, cognitive vulnerability, and heightened inflammatory burden, necessitates a fundamentally different approach to early recovery. Physiologic gating, synchronization with anticoagulation pharmacokinetics, integration of cognitive and motor training, and judicious use of neuromodulation collectively define a modern framework tailored to this population. By embedding rehabilitation within a comprehensive Heart–Brain model, clinicians can address the full spectrum of factors influencing recovery, from cellular neuroplasticity to systemic cardiovascular regulation. Such an approach acknowledges that functional restoration is not achieved in isolation but emerges from the coordinated stabilization and adaptation of interconnected organ systems. As evidence continues to mature, the challenge will be to translate these principles into scalable, reproducible protocols that maintain fidelity to individual patient needs while advancing overall standards of care [39,40,56].

## 8. Limitations of the Study

This review is subject to several limitations inherent to its design. First, as a narrative synthesis rather than a systematic review with meta-analysis, the potential for selection bias regarding the included literature cannot be entirely excluded, despite the application of rigorous inclusion criteria. Second, and most critically, the “Hemodynamic Gating Matrix” and the “Heart–Brain Team” models presented herein constitute theoretical frameworks rather than established clinical standards. These algorithms were developed through the extrapolation of data from separate cardiovascular guidelines and general stroke rehabilitation protocols.

Our comprehensive database search revealed a significant paucity of high-quality Randomized Controlled Trials directly addressing the intersection of specific AF management strategies and early mobilization safety. Consequently, the proposed therapeutic concepts rely on pathophysiological reasoning and the synthesis of indirect evidence rather than high-certainty empirical data. Therefore, these protocols should be interpreted as a conceptual proposal for optimizing care in this specific patient subset, requiring validation in future prospective studies before universal clinical implementation. Finally, the heterogeneity of the geriatric AF population limits the generalizability of these recommendations to all clinical scenarios.

## 9. Conclusions

Atrial fibrillation remains a growing clinical and public health challenge driven by population aging and modifiable cardiometabolic risk factors. Early identification and targeted risk factor management may improve disease control and support primary prevention. Advances in interventional and pharmacological therapies, including novel anticoagulants, aim to optimize safety and efficacy. Effective rehabilitation following AF-related stroke requires an integrated, physiology-driven approach coordinated by a multidisciplinary Brain–Heart Team Model to balance neurological recovery with cardiovascular stability.

## Figures and Tables

**Table 1:** Inclusion and exclusion criteria for literature selection.

Criterion	Inclusion Criteria	Exclusion Criteria
**Study Design**	Randomized Controlled Trials (RCTs), prospective/retrospective cohort studies, systematic reviews, meta-analyses, and official clinical guidelines (e.g., ESC, AHA/ASA).	Case reports, small case series (*n <* 10), editorials, letters to the editor, expert opinions without original data, and conference abstracts lacking full text.
**Thematic Scope**	Studies explicitly addressing the interdisciplinary “Brain–Heart Rehabilitation” axis: AF management coupled with stroke recovery, hemodynamic monitoring during mobilization, or anticoagulation in rehab settings.	Studies focusing exclusively on isolated domains: general electrophysiology, general stroke rehabilitation without AF specificity, or basic science not translated to clinical practice.
**Timeframe and Language**	Full-text articles published in English between 1 January 2020, and 31 January 2026, with priority given to recent data (2023–2026) reflecting current pharmacotherapy standards.	Publications prior to the year 2020 (unless seminal historical references), non-English manuscripts, and duplicate records identified during the initial database screening.
**Subjects and Setting**	Human adult populations diagnosed with atrial Fibrillation (all types) and/or ischemic stroke; studies conducted in clinical, outpatient, or rehabilitation settings.	Animal models (pre-clinical studies), pediatric populations, and studies involving investigational drugs or devices not currently approved for clinical use.
**Outcome Measures**	Measurable clinical endpoints: functional independence (e.g., mRS, Barthel Index), hemodynamic stability parameters, cognitive trajectory, or safety events (e.g., bleeding, recurrent stroke).	Studies lacking defined clinical endpoints, qualitative descriptive reports without measurable outcomes, or articles with insufficient data transparency.

**Table 2:** Key evidence synthesizing modern rehabilitation strategies in AF-related stroke (2020–2025).

Author	Year	Study Type	Population/Sample	Findings and Conclusions
**Fischer U et al.** [39].	2023	Randomized Controlled Trial (RCT)	N = 2013 (acute ischemic stroke with AF)	Early anticoagulation (NOAC within 48 h) was safe and did not increase the risk of intracranial hemorrhage compared to later initiation. Supports “Rehab-Sync”: Allows earlier mobilization under anticoagulant protection.
**Oldgren J et al.** [40].	2022	Registry-Based Randomized Trial	N = 888 (acute ischemic stroke with AF)	Early NOAC initiation (1–4 days) was non-inferior to delayed start (5–10 days) regarding stroke recurrence and bleeding. Validation: Justifies the feasibility of active rehabilitation in the first week post stroke.
**Yu S et al.** [41].	2025	Narrative Review and Conceptual Framework	Review of 21 studies focused on post-stroke hemodynamic management	Proposed a paradigm shift from “one-size-fits-all” BP targets to an individualized, function-oriented strategy. Argues that BP management must optimize neuroplasticity and functional recovery, not just survival, supporting our “Hemodynamic Gating” concept.
**Saglietto A et al.** [42].	2021	Physiological In Vivo Study (NIRS)	N = 53 (AF patients undergoing cardioversion)	Demonstrated that irregular AF rhythm causes “beat-to-beat” variability in cerebral microcirculatory perfusion, leading to critical hypoperfusion events. Crucial Evidence: Provides the mechanistic basis for the hemodynamic instability risk in AF brains.
**Castle-Kirszbaum M et al.** [43].	2022	Systematic Review	N = N/A (review of 48 studies on CO/CBF)	Confirmed the “Cardio-Cerebral Coupling” phenomenon: Reduced cardiac output directly compromises cerebral blood flow in patients with impaired autoregulation. Validation: Justifies checking cardiac reserve before verticalization.
**Aftyka J et al.** [44].	2023	Systematic Review	N = 1305 (aggregate from 36 studies)	Heart Rate Variability (HRV) identified as a robust predictor of stroke course and complications. Low HRV correlates with autonomic exhaustion. Supports “autonomic integrity”: Validates HRV monitoring as a safety tool in rehabilitation.
**Feng Z et al.** [45].	2025	Systematic Review and Meta-Analysis	N = 3,491,423 (aggregate from 39 observational studies)	Identified key modifiable risk factors for cognitive decline (hypertension, diabetes). Confirmed that NOACs (OR = 0.63) and catheter ablation (OR = 0.74) are significantly protective against cognitive impairment. Validation: Strongest evidence for the “Heart–Brain Team” prevention strategy.
**Koh YH et al.** [46].	2022	Systematic Review and Meta-Analysis	N = 2.8 million (global cohort)	AF increases the risk of cognitive impairment by 39% even in the absence of clinical stroke, driven by silent micro-emboli and hypoperfusion. Validation: Justifies the need for routine cognitive screening in all AF patients.
**Wang X et al.** [47].	2023	Meta-Analysis of RCTs	N = 18 RCTs (stroke rehabilitation)	Vagus Nerve Stimulation (VNS) significantly improves upper limb motor function (Fugl–Meyer score) compared to sham. Neuromodulation: Provides evidence base for adjunctive VNS, though safety in AF requires caution (as noted in Discussion).
**Zhang X et al.** [48].	2022	Meta-Analysis of RCTs	N = 22 RCTs (stroke gait training)	Dual-task training significantly improved step length and cadence compared to single-task training. Rehab Strategy: Supports the “Cognitive–Motor Integration” protocol for restoring real-world functionality.

**Table 3:** Operationalization of the Hemodynamic Gating Matrix: protocols, thresholds, and escalation pathways.

Input Data (Monitoring Parameter)	Physiological Readiness Thresholds (Go-Criteria)	Interruption Criteria (Stop-Signal)	Mobilization Escalation Pathway	Evidence Basis (Extrapolation Source)
**1. Chronotropic Competence** (Continuous Telemetry)	Resting Ventricular Rate (VR) < 100 bpm. Absence of new-onset arrhythmia or pauses > 3 s.	Rapid Ventricular Response: Exertional VR increase to >110 bpm. De novo atrial fibrillation onset during session.	Level I: If criteria met → Proceed from supine to head-of-bed elevation (30–45°).	Extrapolated from 2024 ESC Guidelines (lenient rate control limits to preserve diastolic filling) [21,77].
**2. Systemic Perfusion Pressure** (Non-Invasive Blood Pressure—NIBP)	Systolic BP: 120–180 mmHg. Mean Arterial Pressure (MAP): >70 mmHg.	Hypoperfusion Alert: SBP < 90 mmHg or MAP drop > 20% from baseline. Hypertensive Surge: SBP > 180 mmHg.	Level II: If stable at 45° for 5 min → Proceed to Sitting at Edge of Bed (Dangling).	Based on AVERT trial safety analysis (avoiding U-shaped mortality curve) and Yu et al. (2025) individualized BP targets [41,76].
**3. Orthostatic Tolerance** (Active Stand Test/Sit-to-Stand)	SBP delta: <20 mmHg drop. Diastolic BP delta: <10 mmHg drop upon verticalization.	Orthostatic Failure: Sustained SBP drop ≥ 20 mmHg within 3 min. POTS Pattern: HR increase >30 bpm without hypotension.	Level III: If orthostasis is negative → Proceed to Active Standing/Transfer to Chair.	Consensus definition of Orthostatic Hypotension applied to stroke cohorts [75,78].
**4. Autonomic Stability** (Short-Term BPV and Symptomatology)	Coefficient of Variation (SBP): <15%. Borg scale (RPE): <11/20 (light exertion).	Autonomic Storm: Profuse sweating, pallor, or fluctuating alertness. Fatigue: Disproportionate dyspnea or RPE > 13/20.	Level IV: If BPV stable → Initiation of Gait Training/Dual-Task Activity.	Extrapolated from Castle-Kirszbaum (2022) on cardio-cerebral coupling and BPV impact on penumbra [43].

## Data Availability

No new data were created or analyzed in this study.

## Appendix A. Detailed Search Strategy

Databases: PubMed/MEDLINE, Scopus, Cochrane Library.

Final Search Date: 31 January 2026.

Search String (PubMed format): (“Atrial Fibrillation”[MeSH] OR “AF” OR “Atrial Flutter”) AND (“Stroke”[MeSH] OR “Ischemic Stroke” OR “Cerebrovascular Accident”) AND (“Rehabilitation”[MeSH] OR “Early Mobilization” OR “Physical Therapy” OR “Recovery”) AND (“Hemodynamics” OR “Safety” OR “Autonomic”).

Inclusion Filters: Humans, English, Full Text, 2020–2026.
